# Chat GPT Performance in Multi-Disciplinary Boards—Should AI Be a Member of Cancer Boards?

**DOI:** 10.3390/healthcare13182254

**Published:** 2025-09-09

**Authors:** Ibrahim Dogan, Mehmet Kadir Bartin, Ezgi Sonmez, Erdogan Seyran, Halil Alper Bozkurt, Mehmet Yuksek, Ezgi Dicle Serbes, Gunel Zalova, Sebahattin Celik

**Affiliations:** 1 Department of General Surgery, Van Training and Research Hospital, University of Health Sciences, 65300 Van, Türkiye; 2 Department of Medical Oncology, Van Training and Research Hospital, University of Health Sciences, 65300 Van, Türkiye; 3 Department of Radiology, Van Training and Research Hospital, University of Health Sciences, 65300 Van, Türkiye; 4 Department of Pathology, Van Training and Research Hospital, University of Health Sciences, 65300 Van, Türkiye; 5 Department of Radiation Oncology, Van Training and Research Hospital, University of Health Sciences, 65300 Van, Türkiye

**Keywords:** artificial intelligence, multi-disciplinary tumor board, cancer

## Abstract

Background: Multidisciplinary Tumor Councils (MDTs) are vital platforms that provide tailored treatment plans for cancer patients by combining expertise from various medical disciplines. Recently, Artificial Intelligence (AI) tools have been investigated as decision-support systems within these councils. Methods: In this prospective study, the compatibility of AI (ChatGPT-4.0) with MDT decisions was evaluated in 100 cancer patients presented to the tumor council between November 2024 and January 2025. AI-generated treatment recommendations based on anonymized, detailed clinical summaries were compared with real-time MDT decisions. Cohen’s Kappa and Spearman correlation tests were used for statistical analysis. Results: Neoadjuvant treatment (45%) and surgery (36%) were the most frequent MDT decisions. AI recommended surgery (39%) and neoadjuvant treatment (37%) most frequently. A high concordance rate of 76.4% was observed between AI and MDT decisions (κ = 0.764 [95% CI; 0.658–0.870] *p* < 0.001, ρ = 0.810 [95% CI; 0.729–0.868], *p* < 0.001). Most inconsistencies arose in cases requiring individualized decisions, indicating AI’s current limitations in incorporating contextual clinical judgment. Conclusion: AI demonstrates substantial agreement with MDT decisions, particularly in cases adhering to standardized oncological guidelines. However, for AI integration into clinical workflows, it must evolve to interpret real-time patient data and function transparently within ethical and legal frameworks.

## 1. Introduction

Approximately 18.1 million new cancer (CA) cases are diagnosed each year in the world, and 9.6 million people lose their lives due to cancer [1]. The incidence of cancer is increasing worldwide due to aging and negative lifestyle changes [2]. There are various treatment options for cancer patients, and these options are affected by many factors. Many factors, such as patients’ socio-demographic characteristics, comorbidities, lifestyle, and tumor status, are effective when making a treatment decision. In addition, technological developments constantly update diagnosis and treatment options. Therefore, managing cancer patients is very important [3,4]. The treatment plan of cancer patients becomes more difficult due to new drug discoveries, updated scientific guidelines, and rapidly changing evidence. To overcome these difficulties, meetings consisting of physicians from different branches called Multidisciplinary Tumor Councils (MDTs) have been established [5]. Medical and radiation oncologists, radiologists, surgeons, and pathologists usually participate in MDTs and decide on the patient’s most appropriate and effective treatment option [6]. For this reason, MDTs are essential in providing a comprehensive and multidisciplinary approach for each patient and tailoring treatment plans to individual needs [5,7].

Artificial Intelligence (AI) is a computer program that mimics cognitive functions to create systems that learn and think like humans [8]. AI can perform cognitive functions such as perception, reasoning, decision making, problem solving, learning, and interacting with the environment [9]. AI is also frequently used in medicine, particularly in diagnosing and treating diseases [10].

Many studies in MDT have investigated artificial intelligence as a decision-support tool. Studies conducted on breast, lung, stomach, brain, and cervical cancers have determined a high rate of compatibility [6,11,12,13,14,15]. Recent advances in artificial intelligence (AI) have introduced several large language models (LLMs), such as GPT-3.5, GPT-4.0, Bard, and Med-PaLM, which differ in their architectures, data sources, and clinical applicability. However, despite these developments, few studies have evaluated the real-time performance of such models in multidisciplinary tumor board (MDT) decision making. To our knowledge, this is the first prospective study to assess the alignment between ChatGPT-4.0 and MDT treatment decisions in oncology patients. This highlights the potential role of ChatGPT-4.0 as a decision-support tool, particularly in resource-limited clinical settings

Within the scope of this research, we aimed to elucidate the compatibility of artificial intelligence with the decisions made in patients brought to the multidisciplinary tumor council. We sought to answer whether we can accept artificial intelligence as a new member, other than humans, as a decision-support tool in the council.

## 2. Method

### 2.1. Study Design

This prospective study was conducted on 100 patients who attended the tumor council at Van Regional Education and Research Hospital between November 2024 and January 2025. All procedures followed were in accordance with the ethical standards of the responsible committee on human experimentation (institutional and national) and with the Helsinki Declaration of 1975, as revised in 2008. Our institution has granted ethics committee approval on 4 November 2024 with protocol number B.30.2.YYU.0.01.00.00/95. Informed consent has been obtained from all participants. This trial has been registered at ClinicalTrials.gov under the registration number NCT069866564.

The multidisciplinary tumor board is composed of specialists from medical oncology, radiation oncology, nuclear medicine, radiology, pathology, and surgical disciplines. The information of each patient to be presented at the board is shared by the primary physician with all board members one week in advance. During the meeting, the patient’s demographic data, clinical complaints, physical examination findings, comorbidities, performance status, laboratory results, pathology reports, and radiological findings are reviewed by the participants. Imaging studies are presented and re-evaluated in real time by radiology and nuclear medicine specialists using a projector. Following the discussion, a consensus decision is reached based on the opinions of all attending specialists.

During the planning phase of the study, the structure and operational principles of the multidisciplinary tumor board were first introduced to Chat-GPT 4o. It was clearly stated that, after the patients were discussed in the tumor board and treatment plans were determined, the AI’s decision would be queried solely for comparison purposes and would not influence the actual treatment planning.

The data of each patient presented to the board were compiled in a Word document by a physician who did not attend the board meeting and was blinded to the board’s decision. An example patient file was provided as a supplementary document. The file included detailed information such as the patient’s sex, age, comorbidities, performance status, clinical complaints, physical examination findings, radiology and pathology reports, and laboratory results (Table 1).

This document was uploaded to Chat-GPT 4o, and the model was asked to evaluate the patient and propose a treatment plan based solely on the provided data. No additional prompts or questions were given in order to avoid external guidance beyond the shared information.

The decisions made by Chat CPT-4o and the council decisions were coded as neoadjuvant treatment, surgery, radiotherapy, additional examination request, follow-up, adjuvant therapy, interventional-surgical sampling, endoscopic intervention, and palliative. Artificial intelligence decisions were not reported to the council members. An independent statistics expert analyzed all results.

The study’s primary aim was to investigate the tumor council’s and artificial intelligence’s suitability in decision making. The second aim was to determine the reasons for patients’ inconsistent decisions.

### 2.2. Participants

Patients over the age of 18 who were diagnosed with cancer in pathology, regardless of their anatomical location, and were brought to the tumor council, were included in the study. All participants were considered patients brought to the council for the first time. Patients with incomplete information, those without a pathological cancer diagnosis, those brought for radiological opinion, and those brought to two or more times to council were not included in the study.

### 2.3. Statistical Analysis

Patient data collected within the study’s scope were analyzed using the IBM Statistical Package for the Social Sciences (SPSS) for Windows 26.0 (IBM Corp., Armonk, NY, USA) package program. To assess data distribution, we employed the Kolmogorov–Smirnov test to determine whether continuous variables followed a normal distribution or not. Frequency and percentage for categorical data and mean and standard deviation for continuous data were given as descriptive values. The agreement between MDT and AI was measured using Cohen’s Kappa test. Correlation analysis between variables was performed using Pearson or Spearman correlation techniques, as appropriate. For comparisons between groups, the “Independent Sample *t*-test” was used for two groups, and the “Pearson Chi-Square Test” was used to compare categorical variables. The results were considered statistically significant when the *p*-value was less than 0.05.

## 3. Results

A total of 100 patients were included in the study. 48% (*n* = 48) of the patients were male, and 52% (*n* = 52) were female. The mean age was 58.4 ± 12.6 years, ranging from 24 to 86 years (Table 2).

### 3.1. Distribution of Cancer Types

Among the cases presented to the tumor council, the most common malignancies were breast cancer (*n* = 28) with 28% and stomach cancer (*n* = 23) with 23%. These were followed by esophageal (17%, *n* = 17), colon (9%, *n* = 9), cardioesophageal junction (7%, *n* = 7), rectum (6%, *n* = 6), pancreas (2%, *n* = 2), lung (1%, *n* = 1) cancers and other tumors (7%, *n* = 7). Of the decisions taken, 51% were related to stomach or breast cancer (Table 3).

### 3.2. Multidisciplinary Tumor Council (MDT) Decisions

The most frequently preferred treatment approach in multidisciplinary council decisions was neoadjuvant treatment (*n* = 45) at a rate of 45%. This was followed by direct surgery (*n* = 36) at a rate of 36% and additional examination request (*n* = 11) at 11%. Less frequently preferred options included radiotherapy (2%, *n* = 2), adjuvant treatment (1%, *n* = 1), follow-up (1%, *n* = 1), biopsy (1%, *n* = 1), and palliative care (3%, *n* = 3) (Table 4).

### 3.3. Artificial Intelligence (AI) Decisions

AI most frequently suggested direct surgery (*n* = 39) at a rate of 39%, neoadjuvant treatment (*n* = 37) at a rate of 37%, and additional examination (*n* = 12) at a rate of 12%. Other decisions were distributed as follows: radiotherapy (2%, *n* = 2), follow-up (3%, *n* = 3), adjuvant treatment (2%, *n* = 2), endoscopic intervention (1%, *n* = 1), and palliative care (4%, *n* = 4) (Table 4).

### 3.4. Cross-Table Analysis for Decision Consistency

According to the cross-tabulation analysis, the AI also recommended the same decision in 35 of 45 cases (77.8%) where the human council recommended neoadjuvant treatment. In the remaining patients, AI recommended surgery in 8, additional examination in 1, and palliative care in 1. Of these 8 cases, 3 were stomach cancer, 2 were breast cancer, 1 was rectum cancer, 1 was pancreatic cancer, and 1 was esophageal cancer. The MDT recommended neoadjuvant treatment for the pancreatic cancer patient because he was considered inoperable due to vascular invasion. The patient to whom the AI recommended follow-up was the patient with metastatic colon cancer who received neoadjuvant treatment. In the patient who did not respond to treatment, MDT recommended neoadjuvant continuation, while artificial intelligence suggested follow-up. Artificial intelligence suggested palliative treatment to the esophageal ca patient with supraclavicular, paragastric, and celiac lymph node metastasis who was recommended neoadjuvant by MDT. AI also made surgical decisions in 31 of the 36 cases (86.1%) where the human council recommended surgery, and different decisions were made in the others (NEO: *n* = 2, follow-up: *n* = 1, adjuvant: *n* = 1, endoscopic intervention: *n* = 1). One of the patients who decided to have surgery by MDT and was recommended neoadjuvant by AI was an early-stage breast cancer patient with no pathological lymph nodes in the axilla. The other patient was an advanced-stage gastric cancer patient. MDT suggested surgery due to the mass causing obstruction. The patient recommended for adjuvant treatment by AI was a 59-year-old patient who underwent appendectomy due to acute appendicitis, and adenocarcinoma infiltration was detected in the pathology. Detailed examinations revealed a hepatic flexure tumor. The patient underwent right hemicolectomy, and a recurrent mass was detected in the anastomosis line in the Positron Emission Tomography–Computed Tomography (PET-CT). MDT made a surgical decision. While MDT made the surgical decision for the patient with a mass in the esophagus at 36 cm, diagnosed with Squamous Cell Carcinoma (SCC), and no lymph node metastasis, AI recommended Endoscopic Mucosal Resection/ Endoscopic Submucosal Dissection (EMR/ESD) as an endoscopic intervention. Although MDT made the surgical decision for the patient with a giant polyp in the sigmoid colon, AI recommended follow-up (Table 5).

### 3.5. Concordance of Artificial Intelligence and Human Decisions

**Figure 1:**
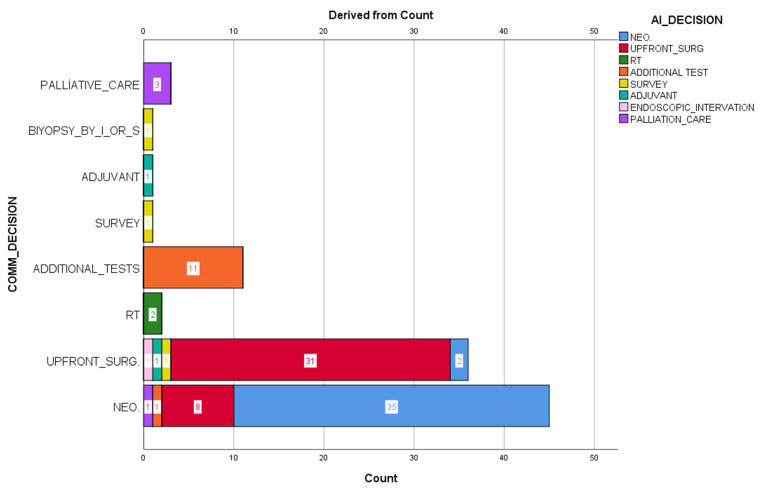
Visualization of Human and AI Decisions.

Cohen’s Kappa value was 0.764 [95% CI; 0.658–0.870], which is statistically significant (*p* < 0.001). In addition, the Spearman correlation coefficient was calculated as 0.810 [ 95% CI; 0.729–0.868] (*p* < 0.001) (Table 6) (Figure 1).

The agreement between the tumor council (COMM_DECISION) decisions and the AI (AI_DECISION) decisions is shown. The graph shows the AI-recommended decisions for each human decision in stacked columns. The highest agreement is seen in neoadjuvant and upfront surgery decisions. The decision differences are particularly concentrated between these two categories.

**Figure 2:**
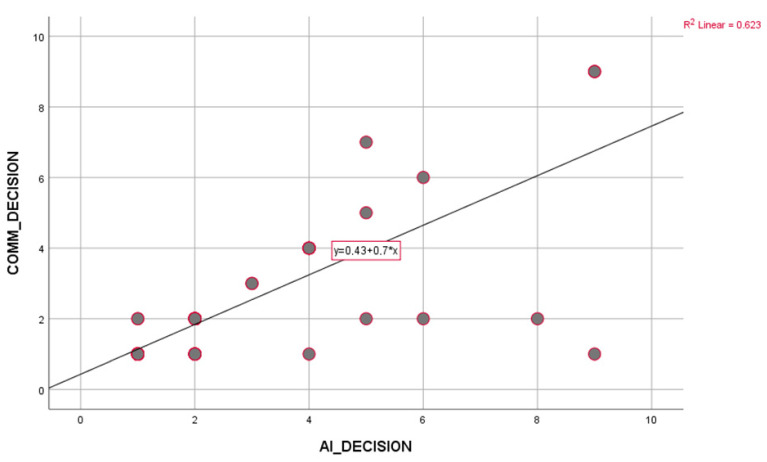
Correlation Between AI and Tumor Council Decisions.

These results show that AI-supported decision systems overlap with the decisions taken in multidisciplinary councils and provide significant agreement, especially in the main treatment decisions (neoadjuvant vs. surgery) (Figure 2).

Scatter plot showing the relationship between AI (x-axis) and tumor council (y-axis) decisions. The linear regression curve and equation (y = 0.43 + 0.7x) with R^2^ value (0.623) are indicated in the graph. This result shows that there is a medium-high level of positive correlation between the two decision-making systems.

## 4. Discussion

In this prospective study, clinical case-based treatment decisions of multidisciplinary tumor councils (MDT) were compared with the recommendations of the large language model ChatGPT, and as a result of the evaluation conducted on 100 patients, a concordance of 76.4% was determined. Unlike most retrospective analyses in this field, the study’s prospective design allowed direct observation of real-time decision processes and a more objective assessment of how much the model overlaps with clinical reality. In this respect, the study makes an essential contribution to the literature evaluating the practical potential of artificial intelligence as a clinical decision support system.

The 76.4% concordance obtained reveals ChatGPT’s high familiarity with oncological clinical guidelines and standard protocols. The artificial intelligence model made similar recommendations to MDT, especially in cases with clear staging systems, classical treatment algorithms, and low comorbidity burden. Recent studies have similarly reported that large language models can perform strongly in answering medical questions and defining clinical protocols [16,17].

However, a detailed examination of the remaining 23.6% of non-compliance revealed the limitations of ChatGPT. Non-compliance mainly occurred in cases where patient-specific individual factors were not considered. In MDT meetings, clinicians’ decisions are not limited to medical guidelines but also include many variables such as the patient’s general condition, quality of life, comorbidities, potential for treatment compliance, psychosocial factors, and patient preferences. Language models such as ChatGPT, on the other hand, are inadequate in evaluating such contextual information. This shows that artificial intelligence cannot yet be considered a decision maker with real clinical intuition and ethical values. Another limitation of the model’s recommendations is the lack of transparency. ChatGPT does not present to the user what data or information it bases its recommendations on. This situation can create a security gap for clinicians, especially in sensitive patient-specific decisions. This situation, referred to as the “black box” problem in the literature, raises the question of who will assume clinical responsibility for artificial intelligence systems [18]. As long as the ethical and legal responsibility for clinical decisions still lies with human physicians, such support systems should remain in a position of suggestion providers rather than decision makers [19,20]. Nevertheless, large language models such as ChatGPT have significant advantages. The model can be valuable in providing physicians with rapid access to literature, summarizing existing treatment guidelines, evaluating possible alternative treatment options, and providing reference points in treatment planning. In addition, in healthcare systems under time pressure, especially in low-resource regions, it is possible for such AI-supported systems to play a supporting role in clinical decision-making processes by facilitating access to information.

The prospective design of our study allowed us to observe the differences between AI and human decisions promptly. This made it possible to compare decisions theoretically and in practical application conditions. For example, in some cases, the MDT made individualized decisions that deviated from established guidelines, based on factors such as the patient’s likelihood of adhering to treatment or recent concerns regarding quality of life. In contrast, ChatGPT generated recommendations without taking these nuanced, patient-specific considerations into account. A study examining the perspective of surgeons, medical and radiation oncologists on AI during the diagnosis, treatment, and follow-up phases of cancer patients was conducted by Valerio et al. [21]. This study found that surgeons increased their performance and training by assisting the surgeon before, during, and after surgery. It has been stated that medical oncologists benefit from artificial intelligence in molecular profiling and treatment selection, predictive modeling for drug response and personalized therapy, and integration of artificial intelligence in clinical trial design and patient records. They emphasized that radiosonde oncologists receive support from artificial intelligence in the areas of artificial intelligence-supported radiotherapy workflow and prediction of radiotherapy outcomes and toxicity, and that this support reduces workload and increases work quality.

Kim et al. [11] found 92.4% concordance between MDT and artificial intelligence in a study of 405 lung cancer patients in 2018. They determined this rate as 100%, especially in advanced metastatic lung cancers. Benedick et al. [22] found 96% concordance in metastatic patients and 86% concordance in recurrent or metastatic head and neck cancer patients. They concluded from the study that artificial intelligence is mostly an auxiliary tool, needs to be approved by an experienced clinician due to a lack of transparency, and sometimes suggests treatment methods not in the current guidelines. In another study, Benedick et al. [13] investigated the compliance rate of patients with head and neck cancer who were taken to MDT by working on different versions of artificial intelligence. The researcher who used ChatCPT-3.5 and ChatCPT-4.0 found a high level of similarity in both. However, when looking at treatment options, it was seen that, although MDT offered at most two options, ChatCPT-3.5 offered more options than MDT and fewer than ChatCPT-4.0. It was also stated that ChatCPT-4.0 had better summarization, explanation, and clinical recommendations, and references from current literature were provided. On the other hand, he avoided making definitive recommendations and stated that he did not intend to give medical advice or replace a medical doctor.

In the study conducted by Park et al. [12] on 322 gastric cancer patients, they found an agreement between MDT and artificial intelligence of 86%. When they looked at the stages of stomach cancer one by one, they found it to be 96.93% for stage 1, 88.89% for stage 2, 90.91% for stage 3, and 45.83% for stage 4. They stated that artificial intelligence plays an effective role in managing stomach cancer patients in MDT and can even participate as a member of MDT2.

In a retrospective study conducted by Somashekhar et al. [15] on breast cancer patients, they found high rates of concordance, such as 97% and 95% in patients with stage 2 and stage 3 cancer. There was less concordance in patients with stage 1 and stage 4, 80% and 86%. Since the years 2014–2016, when MDT examined the patients, and 2016, when the artificial intelligence examined the same patient information, were not taken into account, the similarity rate was 73%. A blind secondary review was conducted by MDT in 2016 for patients with different results, and the concordance increased from 73% to 93%. When they also examined the concordance rate by age, it was determined that it decreased as age increased, except for patients under 45 and 55–64. It was also determined that concordance decreased much more in patients over 75. Unlike the abovementioned studies, our study evaluated all cancers presented to the council. The high level of agreement in other studies assessing specific cases may be due to the lack of absolute case heterogeneity. The current research seems more realistic in this respect.

A comparative summary of these studies and their reported limitations is presented in Table 7.

### 4.1. Limitations

This study has certain limitations. First, the ChatGPT model cannot fully access the most up-to-date versions of clinical protocols due to the information limits and historical data it was trained on. In addition, since the model cannot directly analyze specific clinical data belonging to the patient, the recommendations remain more general. It should not be forgotten that MDT decisions also carry a certain degree of subjectivity. The study is single-centered, and different results may be obtained when a similar analysis is performed in institutions with varying hospital structures. Not knowing which decision is more effective in the long term does not mean that perfect compliance yields good results. It may be more appropriate to follow how the course progresses, especially in cases of incompatibility. The relatively small sample size (*n* = 100) may limit the generalizability of our findings. However, as this is the first feasibility study evaluating AI-assisted decision making in oncology MDTs, the dataset provides valuable initial insights and guides future multicenter studies with larger cohorts

### 4.2. Recommendations for Future Studies

Future studies should include similar prospective comparisons of different AI models (e.g., Med-PaLM, BioGPT, Claude Medical). In addition, technical developments should be evaluated to integrate models with real patient data (laboratory results, radiology findings, genomic data) to provide contextual recommendations. Pilot applications where clinical decision processes are carried out with real-time AI-supported platforms will also significantly contribute to this literature.

## 5. Conclusions

This prospective study evaluated how much the large language model ChatGPT overlaps with the treatment decisions made by multidisciplinary tumor councils (*MDTs*) on 100 oncology patients. The 76.4% agreement rate shows that AI can successfully mimic guideline-based clinical reasoning in specific standard oncological scenarios. ChatGPT’s decisions largely overlapped with MDTs, especially in patients with precise staging and protocol-based treatments. AI can be a powerful decision support tool in providing guideline information, summarizing alternative treatment options, and facilitating literature access. It can play a significant supporting role in terms of providing rapid access to information, especially in clinical settings with limited resources. Qualitative evaluation of discordant cases revealed that most differences arose in situations requiring individualized clinical decisions. While MDT decisions considered patient-specific contextual factors such as comorbidities, surgical risks, and quality of life, ChatGPT-4.0 primarily relied on standard guideline-based approaches. This highlights the model’s current limitations in interpreting patient-specific nuances.” The integration of AI into MDT decision-making processes will only be possible if it reaches the capacity to analyze real-time patient data, transparently base its recommendations, and align with the human-centered medical approach. Therefore, AI technologies must be further evaluated from ethical, legal, and clinical perspectives and supported by multicenter, comprehensive, advanced studies.

## Figures and Tables

**Table 1:** Case Presentation to Chat-GPT, An Example.

Parameter	Details
Age/Sex	65-year-old male
Presenting Complaint	Dysphagia
ECOG Performance Status	1
Comorbidities	None
Endoscopic Findings	Ulcerovegetative mass from below the Z-line extending 3–4 cm into the cardia
Histopathological Findings	Malignant epithelial tumor
Immunohistochemistry	PanCK(+), p53 (strong, diffuse+), CD3(-), CD20(-), CD56(+), chromogranin(+), synaptophysin(-), Ki-67: 90%
Preliminary Diagnosis	Mixed adenoneuroendocrine carcinoma (MANEC) suspected; final dx pending surgical specimen
Thoracic CT	4 mm subpleural polygonal subsolid nodule in right upper lobe
Abdominal CT	Gastric cardia wall thickening; lymphadenopathies; suspected omental cake (not confirmed)
PET CT	None
Laboratory Tests	WBC: 8 × 10^9^/L, Hb: 11 g/dL, Biochemistry: Normal, ELISA: Negative
Tumor Markers	CA 19-9: 19 U/mL, CEA: 1.9 ng/mL

**Table 2:** Gender and Age Distribution of Patients (*n* = 100).

Variable	Category/Statistic	Value
Gender	Male	48 (48.0%)
	Female	52 (52.0%)
Age	Mean ± SD	58.4 ± 12.6
	Median	58
	Range (Min–Max)	24–86

**Table 3:** Distribution of Cancer Types (*n* = 100).

Cancer Type	Frequency	Percent
Esophagus CA	17	17.0%
Cardia CA	7	7.0%
Gastric CA	23	23.0%
Rectum CA	6	6.0%
Colon CA	9	9.0%
Breast CA	28	28.0%
Lung CA	1	1.0%
Pancreas CA	2	2.0%
Others	7	7.0%

**Table 4:** Comparison of MDT and AI Decisions.

Decision Type	MDT (*n*)	MDT(%)	AI Decision (*n*)	AI Decision (%)
Neoadjuvant	45	45.0%	37	37.0%
Upfront Surgery	36	36.0%	39	39.0%
Radiotherapy	2	2.0%	2	2.0%
Additional Tests	11	11.0%	12	12.0%
Survey	1	1.0%	3	3.0%
Adjuvant	1	1.0%	2	2.0%
Biopsy	1	1.0%	1	1.0%
Palliative Care	3	3.0%	4	4.0%

**Table 5:** Cross-Tabulation of Tumor Council and AI Decisions.

MDT Decision/AI Decision	NEO	Upfront Surgery	RT	Additional Test	Survey	Adjuvant	Endoscopic Intervention	Palliation Care
Neo	35	8	0	1	0	0	0	1
Upfront Surgery	2	31	0	0	1	1	1	0
RT	0	0	2	0	0	0	0	0
Additional Tests	0	0	0	11	0	0	0	0
Survey	0	0	0	0	1	0	0	0
Adjuvant	0	0	0	0	0	1	0	0
Biopsy	0	0	0	0	1	0	0	0
Palliative Care	0	0	0	0	0	0	0	3
Total	37	39	2	12	3	2	1	4

**Table 6:** Statistical Measures.

Measure	Value	Standard Error	*p*-Value
Spearman Correlation	0.810	0.055	<0.001
Cohen’s Kappa	0.764	0.054	<0.001

**Table 7:** Comparative summary of previous and recent studies evaluating AI-assisted oncology MDT decision support, including reported limitations and performance metrics.

Study/Year	AI Model	Design	Sample Size	Cancer Types	Reported Limitations	Performance/Agreement (%)	How AI was Addressed in This Study
Kim et al., 2018 [11]	IBM Watson	Retrospective	405	Breast & lung cancers	Limited access to patient-specific data; outdated clinical guidelines	72%	Used updated structured summaries; real-time data integration remains limited
Park et al., 2022 [12]	IBM Watson	Prospective	322	Colorectal cancer	Small sample size; no qualitative discordance analysis	75%	Qualitative evaluation of discordant cases included
Benedick et al., 2023 [22]	GPT-3.5 vs. GPT-4	Retrospective	150	Various solid tumors	Lack of real-time clinical data; limited external validation	GPT-3.5: 68%/GPT-4: 74%	Prospective data collection improved applicability

## Data Availability

The data supporting this study’s findings are available upon request from the corresponding author. **Use of Artificial Intelligence:** The authors used AI and AI-assisted Technologies (Grammarly and MS Word Editor) in the writing process. These technologies improved the readability and language of the work. Still, they did not replace key authoring tasks such as producing scientific or medical insights, drawing scientific conclusions, or providing clinical recommendations. The authors are ultimately responsible and accountable for the content of the whole work.
